# Integration and fusion of standard automated perimetry and optical coherence tomography data for improved automated glaucoma diagnostics

**DOI:** 10.1186/1471-2415-11-20

**Published:** 2011-08-04

**Authors:** Dimitrios Bizios, Anders Heijl, Boel Bengtsson

**Affiliations:** 1 Department of Clinical Sciences Malmoe, Ophthalmology, Skåne University Hospital, Lund University, SE-205 02 Malmoe, Sweden

## Abstract

**Background:**

The performance of glaucoma diagnostic systems could be conceivably improved by the integration of functional and structural test measurements that provide relevant and complementary information for reaching a diagnosis. The purpose of this study was to investigate the performance of data fusion methods and techniques for simple combination of Standard Automated Perimetry (SAP) and Optical Coherence Tomography (OCT) data for the diagnosis of glaucoma using Artificial Neural Networks (ANNs).

**Methods:**

Humphrey 24-2 SITA standard SAP and StratusOCT tests were prospectively collected from a randomly selected population of 125 healthy persons and 135 patients with glaucomatous optic nerve heads and used as input for the ANNs. We tested commercially available standard parameters as well as novel ones (fused OCT and SAP data) that exploit the spatial relationship between visual field areas and sectors of the OCT peripapillary scan circle. We evaluated the performance of these SAP and OCT derived parameters both separately and in combination.

**Results:**

The diagnostic accuracy from a combination of fused SAP and OCT data (95.39%) was higher than that of the best conventional parameters of either instrument, i.e. SAP Glaucoma Hemifield Test (p < 0.001) and OCT Retinal Nerve Fiber Layer Thickness ≥ 1 quadrant (p = 0.031). Fused OCT and combined fused OCT and SAP data provided similar Area under the Receiver Operating Characteristic Curve (AROC) values of 0.978 that were significantly larger (p = 0.047) compared to ANNs using SAP parameters alone (AROC = 0.945). On the other hand, ANNs based on the OCT parameters (AROC = 0.970) did not perform significantly worse than the ANNs based on the fused or combined forms of input data. The use of fused input increased the number of tests that were correctly classified by both SAP and OCT based ANNs.

**Conclusions:**

Compared to the use of SAP parameters, input from the combination of fused OCT and SAP parameters, and from fused OCT data, significantly increased the performance of ANNs. Integrating parameters by including a priori relevant information through data fusion may improve ANN classification accuracy compared to currently available methods.

## Background

Glaucoma is an optic neuropathy resulting in characteristic visual field defects. Investigating the relationship between development of functional damage in the visual field and structural glaucomatous changes of the retinal nerve fiber layer (RNFL) has been the purpose of numerous studies [1-5].

Diagnostic instruments providing quantitative analyses in glaucoma assess either functional or structural aspects of the disease. Imaging and quantitative analysis of RNFL measurements can be accomplished with Optical Coherence Tomography (OCT). OCT is a noninvasive interferometric technique that provides cross sectional images and thickness measurements of the RNFL (RNFLT) with high resolution [6] and good reproducibility [7-9]. Standard Automated white-on-white Perimetry (SAP) is the standard for examining the visual field. Perimetric tests are able to provide quantitative measurements of differential light sensitivity at many test point locations in the visual field, and commercially available statistical analysis packages help clinicians in identifying significant visual field loss [10,11]. The diagnostic performance of both OCT and SAP in glaucoma as well as the correlation between SAP and OCT measurements has been investigated [12-15].

It is conceivable that integration of functional and structural test measurements could provide more relevant information and thus improved diagnostic performance for classification systems when used as input data. The relevance of integrated diagnostic information is dependent on the underlying relationship between structural and functional measurements. Statistical approaches such as the linear model constructed by Hood et al related RNFLT values to sensitivity losses in SAP [16]. Other studies trying to map the individual visual field test points in SAP to areas of the peripapillary RNFL through different models, showed moderate correlations between visual field sensitivity values and structural measurements [17,18]. More recent attempts to model the function - structure relationship in glaucoma demonstrated that machine learning algorithms, such as radial basis function artificial neural networks (ANNs), improved the modelling accuracy compared to linear methods [19].

The use of machine learning classifiers (MLCs) in glaucoma diagnosis using either functional or structural measures has been previously explored [20]. MLCs like ANNs have been used for classification of tests based on structural or functional measurements [21-28] and for detection of glaucoma progression [29,30]. ANN-based classification demonstrated better accuracy than linear methods [23,24,31] and performed at least as well as human experts [32].

Recent attempts to provide a combined evaluation of structural and functional tests showed promising results [33,34], though few studies have examined the diagnostic performance of combining functional and structural data with MLCs for glaucoma diagnosis [35,36]. One of the main advantages of MLCs is their ability to learn a classification task by training on given examples. Such adaptive classification based on the available data is useful, since a complete analytic theory of the structure-function relationship in glaucoma does not yet exist. The performance of MLCs can be influenced by a number of factors including data selection bias, choice of input and classifier architecture.

The purpose of this study was to investigate whether the integration of information from SAP and OCT data could improve the accuracy of glaucoma diagnosis, by using the data as input in ANN based classifiers. We evaluated the performance of simple combination of OCT and SAP data as well as novel approaches based on data fusion by utilizing á priori knowledge about the physiologic relationship between the RNFL and visual function in glaucoma.

## Methods

This study is based on analysis of prospectively collected data from randomly selected healthy individuals from a defined catchment area and glaucoma patients followed at the Department of Ophthalmology at Skåne University Hospital, Malmö Sweden. The study was conducted according to the tenets of the Declaration of Helsinki and was approved by the Regional Ethical Review Board of Lund, Sweden. All healthy individuals and clinical glaucoma patients included in the study provided informed consent prior to any examinations.

### Healthy Individuals

We performed a random selection from a population register containing 4,718 persons over 50 years, living in two primary care catchment areas of Scania, Sweden. This selection yielded a sample of 307 individuals who were invited to participate in the study. Of those, 170 individuals accepted the invitation and underwent a comprehensive ophthalmic examination.

### Clinical Glaucoma Patients

We randomly selected 397 patients with a diagnosis of primary open angle glaucoma, normal tension glaucoma or pseudo-exfoliation glaucoma, from a register of 2,174 visits of patients having these diagnoses, followed at the Department of Ophthalmology, Malmö University Hospital, Sweden between January 2^nd ^2007 and March 13^th ^2008. After review of the 397 patient medical records we excluded patients with any history or additional diagnoses of ocular or systemic pathology affecting the visual field or the RNFL except glaucoma (e.g. neurological disorders or retinal disease). Our reference for the diagnosis of glaucoma was based on optic nerve head (ONH) topography and/or examination of available ONH photographs. After application of our exclusion criteria, 164 patients that fulfilled our diagnostic reference for glaucoma were invited to participate and underwent an ophthalmic examination.

### Examinations

Upon examination a detailed medical history was taken, including current medical conditions and treatments. Individuals with systemic or non-glaucomatous ocular diseases that could affect the ONH, RNFL and visual field were excluded. Persons with lens opacities or intraocular lenses were not excluded from the study. One randomly selected eye from each eligible healthy individual was chosen for inclusion in the study. For glaucoma patients only the affected eye was included in the study. In patients with bilateral disease, the eye with the best Mean Deviation value (i.e. the less negative value denoting milder glaucomatous damage) on the most recent SAP examination was chosen.

The clinical ophthalmic examination consisted of the following parts:

1. Visual acuity was measured using an autorefractor (Humphrey model 595 - Carl Zeiss Meditec, Dublin, CA, USA). Manual refraction was performed when the autorefractor-measured visual acuity values were < 0.8. All participants were required to have visual acuity ≥ 0.5 and refractive error ≥ 5 dioptres (D) sphere and < 3 D cylinder in order to be included in our subsequent analyses.

2. Intraocular pressure was measured by a Goldmann applanation tonometer.

3. Fundoscopy was performed by a trained clinician with a slit-lamp biomicroscope after the use of mydriatic agents (tropicamide 0.5% and phenylephrine hydrochloride 2.5%).

All examined individuals underwent a battery of functional and imaging tests, including:

1. Standard Automated Perimetry (SAP) with the Humphrey Field Analyzer (Carl Zeiss Meditec, Dublin, CA, USA) using the 24 - 2 SITA Standard program. Healthy participants underwent a second SAP examination of the study eye and the results of the second test were subsequently chosen. Perimetric tests were required to have reliable fixation as assessed by the perimetrist and < 15% of false positive answers to be included.

2. Time domain OCT examination after pupil dilation with Stratus OCT (Carl Zeiss Meditec, Dublin, CA, USA) using the Fast RNFL thickness protocol, which derives the RNFLT values by averaging three 3.4 mm circumpapillary scans, each with 256 measurement points (A-scans). All included OCT tests were required to be of good quality as defined by the manufacturer specifications (signal strength > 5) and free of obvious artifacts from incorrect delineation of the RNFL by the instruments segmentation algorithm. The same experienced ophthalmic photographer performed all OCT examinations.

Inclusion of tests from healthy individuals and glaucoma patients in further analyses was based on evaluation of the optic disc during fundus examination. Among healthy individuals only subjects having a normal appearance of the optic disc were included. For inclusion of tests from the glaucoma patients, the eligibility criteria of a glaucomatous optic disc described on their records and/or present in previous optic disc photographs, had to be confirmed during fundus examination.

### Structural and functional test Parameters

Our analyses in this study are based on the following OCT and SAP parameters:

#### OCT RNFLT parameters

##### • RNFLT standard parameters

The StratusOCT RNFL analysis printout provides average thickness measurements for the whole scan circle, the four quadrants and the 12 clock hour sectors of each scan, while highlighting the values that fall below the 5% and 1% significance level, based on comparison to the instrument's normative database. Diagnostic accuracy of the best performing from these parameters was compared to ANN classification performance.

##### • A-scan measurements and PCA processing

We used the 256 averaged A-scan values of the 3 peripapillary scan circles for each OCT test, after we decreased their complexity by means of principal component analysis (PCA). We adjusted PCA to maintain 99.9% of the variation in the data. This was achieved by the first 22 principal components, which were then used as input to the ANN classifiers. All OCT RNFLT data were corrected for age and refractive status (spherical equivalent) based on a separate normative database [37]. We have previously treated the use of A-scan derived parameters as input in automated classifiers [25].

##### • Fused OCT parameters

**Figure 1:**
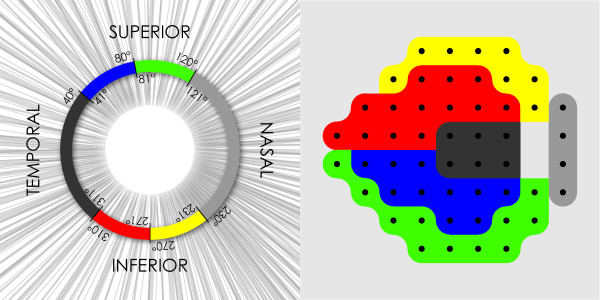
**Map representing the relationship between Standard Automated Perimetry visual field sectors and sections of the peripapillary OCT scan circle**. This map is based on the work of Garway-Heath et al and shows the correspondence between areas of the visual field and peripapillary retinal nerve fiber layer due to the anatomical configuration of the retinal nerve fiber bundles.

The fused OCT parameters were derived by weighting the OCT A-scan measurements of each test with the corresponding scored pattern deviation (PD) values from SAP. In the fusion process for the OCT data we used the map constructed by Garway-Heath et al [38] to represent the relationship between RNFLT of OCT scan circle sectors and differential light sensitivity in specific areas of the visual field, and divided the OCT scan circle and the SITA standard 24-2 SAP test points into 6 sectors accordingly (Figure 1). In each OCT sector and for every A-scan position, we calculated the distribution of RNFLT values based in a separate normative database described elsewhere [37]. The probability values for each age-and refraction corrected OCT A-scan measurement of our test dataset, were then calculated. A-scan values falling below the fifth percentile of the distribution in our normal reference material were transformed through multiplication with an exponential factor. This factor was constructed by calculating the average pattern deviation probability scores (i.e. the sum of all pattern deviation scores divided by the number of SAP test points) of the visual field sector corresponding to each of the OCT scan circle sectors. The fused A-scan values depended on the decrease in RNFLT for the specific A-scan position relative to the distribution of the normative reference material, and the status of the visual field sector corresponding to that location. PCA was subsequently applied on the fused OCT A-scan values. In order to simplify the comparison between non-fused and fused OCT data we included the principal components that retained the same level of variation in the data (99.9%) as in the processing of the previously described non-fused A-scan measurements. In this way PCA provided 38 principal components that were then used as input to the ANN classifiers.

#### SAP test parameters

##### • Glaucoma Hemifield Test (GHT)

The GHT index is available in the standard analysis printout of SAP tests. It is an expert system that classifies SAP tests as within normal limits, borderline or outside normal limits, based on the differences of PD values between test points in mirror image areas of the upper and lower hemispheres of the visual field. We measured the specificity and sensitivity of GHT and compared it to the ANN classifiers.

##### • Pattern deviation probability scores from each of the 52 SAP test point locations (52 parameters)

For each SAP test point, we provided numerical values to the pattern deviation probability map values using a probability scoring scale identical to that used in calculating the GHT [39]. We have previously demonstrated the performance benefits of using pattern deviation probability scores as input to ANNs [21].

##### • Fused SAP parameters (52 parameters)

Fused SAP parameters were derived by weighting each SAP PD scored value with the corresponding OCT A-scan measurements. In the fusion process for SAP data the OCT scan circle and the SITA standard 24-2 SAP test points were divided into six sectors based on the map by Garway-Heath et al [38] (Figure 1). For every visual field sector, the pattern deviation probability score at each test point was transformed by an additive factor. This factor was derived from the age- and refraction-corrected A-scan measurements of the corresponding OCT sector. All A-scan measurements were identified in each OCT sector, and their probability values were assigned a score according to significance level of the deviation from the values of our separate normative database. The probability scoring scale was similar with that used in the calculation of the GHT [39]. The lowest scored probability below the fifth percentile or the highest scored probability above the ninetyfifth percentile of our normal RNFLT distribution from each OCT sector was used as the factor in the fusion process. The fused SAP parameters were obtained by adding this factor to the SAP pattern deviation probability score of each SAP test point in the corresponding visual field sector. In the event that both high and low scored probability values (outside the fifth or ninetyfifth percentile of our normative RNFLT database) existed in the same OCT sector, only the low value was used in the summation process. The fused SAP measurements thus depended on both the status of the visual field sector reflected by the pattern deviation probability scores, and the thickness of the corresponding OCT sector. Visual field defects as indicated by the pattern deviation probability scores could be either accentuated or attenuated during the fusion procedure, depending on the factor of scored probability from the corresponding OCT sector.

**Figure 2:**
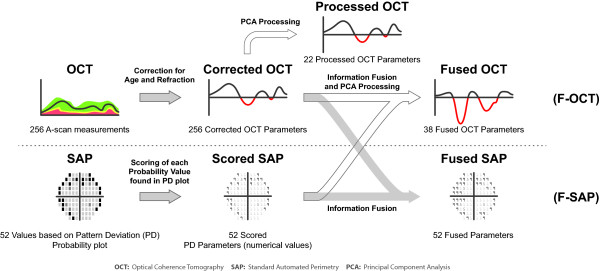
**Examined types of test data used as input to artificial neural network classifiers**. Input to the ANNs consisted of the 22 parameters derived from PCA preprocessing of the OCT A-scan data (processed OCT), the 52 scored SAP parameters, 52 fused SAP parameters derived from incorporation of information from the corrected OCT data, and the 38 fused OCT parameters derived from incorporation of scored SAP data and PCA preprocessing.

The ANNs were trained and tested using the perimetric pattern deviation probability scores and the age-and refraction corrected OCT A-scan measurements after PCA preprocessing, as well as the fused SAP and OCT parameters (Figure 2). We also evaluated the integration of the above parameters by simply combining them. We thus tested:

• Integration of the non-fused SAP and OCT parameters (74 parameters), and

• Integration of the fused OCT and SAP parameters (90 parameters)

### Artificial Neural Networks (ANN)

Our ANN classifier consisted of an ensemble of thirty-five fully connected cascade-forward Multi Layer Perceptrons (MLPs). The number of neurons in the input layer of each cascade-forward MLP was equal to the number of parameters used as input data. All MLPs consisted of 2 hidden layers with tangent hyperbolic transfer functions and an output layer of one neuron with a logistic transfer function that provided the MLP output. The number of neurons in the hidden layers was chosen based on the type of input used in order to achieve the best performance as judged by the results derived from the 10-fold cross validation procedure. Our ANNs were constructed with the MATLAB neural network toolbox version 7 (The MathWorks Inc, Natick, MA, USA) and trained with the scaled conjugate gradient algorithm described by Møller [40].

#### Training the Artificial Neural Networks

ANNs were trained and tested with the 10-fold cross-validation procedure, to reduce bias from training and testing on the same individuals, while fully utilizing our data set. Data were randomly divided into ten subsets, each containing test data from an approximately equal proportion of glaucoma patients and healthy individuals. One subset was used to test classification performance while the remaining nine subsets were used for training purposes. In our ANN ensemble, one out of the nine training subsets was reserved for early stopping of the ANNs in order to avoid overfitting. We additionally used bagging [41] of the remaining eight subsets to create the training sets used by the ANN ensemble. During training, this process was iterated, each time using a different subset as the early stopping set, until all the data subsets had been used to both train and stop the training of the ensemble. We further iterated the training process using each time a different test subset, so that all data could be used both for training and testing the classifiers, and averaged the test results in order to produce a single performance measure for each ANN.

#### Analyses

To measure the classification performance of the ANN ensemble and the diagnostic ability of the compared parameters, we calculated the area under the ROC curve (AROC). The cut off values for all ANNs were calculated based on the best performing specificity-sensitivity pairs (i.e. pairs that provided the largest area under ROC when their values were multiplied) from the 10-fold cross-validation procedure. Significance testing between the AROCs was conducted with DeLong's non-parametric method [42]. We evaluated the agreement in classification between the OCT and SAP based ANNs by calculating odds ratios, which in this case signified the odds that tests classified by the SAP-based ANN receive the same classification by the OCT-based ANN, based on the same classification threshold of 0.5 for both OCT and SAP based classifiers.

The Chi square test was used to find significant differences in the distribution of gender between the healthy individuals and patients with glaucoma, whereas the Mann-Whitney test was used for the continuous variables age, visual acuity and refractive error. Diagnostic accuracy of the SAP and OCT parameters was compared using the McNemar test for correlated proportions.

## Results

Thirty-four healthy individuals and 36 glaucoma patients were excluded because of ophthalmic, neurological and metabolic disorders affecting the visual field and/or the retina, refractive errors and visual acuity outside the defined range for inclusion, erroneous estimation of the RNFL by the OCT segmentation algorithm, and due to inability to complete the examination. Only three healthy persons were excluded based on optic disc criteria (one with optic disc drusen, one with an optic disc hemorrhage and and one with a peripapillary membrane). One patient with glaucoma but normal ONH appearance was also excluded. After application of all exclusion and inclusion criteria, OCT and SAP data from 125 healthy individuals and 135 patients with glaucoma were used in our analyses. Eight of the 135 glaucoma patients were initially part of the healthy population invited to participate in the study, but were diagnosed with glaucoma during the clinical examination and were included in the glaucoma group. The diagnosis for these 8 patients was based on a second clinical examination that included imaging of the ONH and RNFL, SAP testing and evaluation of the fundus and ONH. All OCT tests were of good quality, with mean (±SD) signal strength of 9.95 (±0.23) and 9.36 (±0.97) for the healthy and glaucoma groups respectively. Based on the Mean Deviation of the SAP visual fields, the glaucoma group consisted of 49 patients (ca 36%) with early, 32 patients (ca 24%) with moderate and 54 patients (ca 40%) with advanced glaucomatous visual field loss. The demographic characteristics of the healthy subjects and glaucoma patients can be seen in Table 1. The significantly lower visual acuity of the glaucoma group could be attributed to the higher incidence of lens opacities in this group.

**Table 1:** Demographic data of included healthy subjects and patients with glaucoma.

	Healthy (n = 125)	Glaucoma (n = 135)	p - value (Mann-Whitney test)
**Gender** (female/male)	66/59	79/56	NS*(χ^2 ^test)

**Age** (years)	64.65 ± 8.11	73.36 ± 7.81	< 0.0001

**Visual Acuity** (decimal scale)	1.00 ± 0.15	0.86 ± 0.19	< 0.0001

**Refractive error** (spherical equivalent)	+0.53 ± 1.74	-0.15 ± 1.82	0.0015

**Visual Field** (MD)†	-0.66 ± 1.77	-11.04 ± 8.21	< 0.0001

All values except for gender are represented as mean ± standard deviation.

* NS: non significant

† MD: mean deviation value in decibel, as measured by 24-2 SITA Standard program

The integration of fused data offered higher diagnostic accuracy compared to the best performing SAP and OCT algorithms that exist in the available analysis packages of each instrument. For SAP, the GHT (with borderline results signifying glaucoma) provided an accuracy of 86.92%. For OCT, RNFLT abnormally depressed in at least one quadrant at the 5% significance level, had an accuracy of 91.54%. The combination of non-fused data led to a diagnostic accuracy of 93.85%, significantly better than GHT (McNemars test: p = 0.006), whereas the accuracy of combined fused data was 95.39%, higher than the accuracy of both the GHT (McNemars test: p < 0.0001) and OCT RNFLT algorithm (McNemars test: p = 0.031).

**Figure 3:**
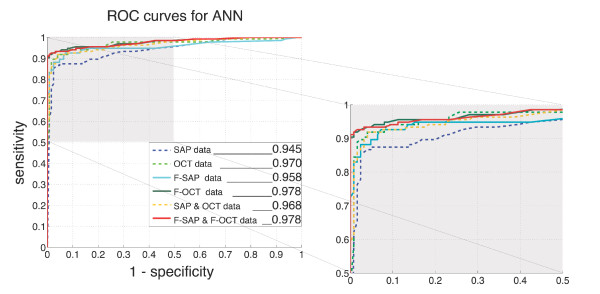
**Performance measured as Area Under the Receiver Operating Characteristic Curve (AROC) for the compared parameters**. Artificial Neural Network (ANN) AROCs for the different input types used. The upper quadrant of the diagram (shaded area) is shown in magnification. The largest AROCs were created by Artificial Neural Network (ANN) ensembles with input based on the fused OCT data and the combined fused OCT and SAP data. Figure abreviations: SAP data: Standard Automated Perimetry data, based on Pattern Deviation (PD) probability scores. F-SAP data: Fused SAP data, based on weighted transformation of PD probability scores with OCT-derived probability scores. OCT data: Age- and refraction corrected OpticalCoherence Tomography A-scan data, optimized by principal component analysis (PCA). F-OCT data: Fused OCT data, based on weighted transformation of A-scan measurements with PD probability scores and optimized by PCA.

The two ANNs with input based on the fused OCT and the combined fused OCT and SAP data respectively provided almost identical AROC values of 0.978, performing significantly better than the ANN based on the SAP measurements alone. Utilizing input based on the combined non-fused OCT and SAP measurements did not lead to similar significant improvements. The AROCs of ANNs based on the fused and non-fused parameters are shown in Figure 3. Significance testing between the compared parameters is shown in Table 2.

**Table 2:** Performance Comparison between Artificial Neural Networks based on fused, combined and single types of data.

	F-SAP data (AROC:0.958)	F-OCT data (AROC:0.978)	SAP & OCT Data (AROC:0.968)	F-SAP & F-OCT data (AROC:0.978)
SAP data (AROC: 0.945)	0.502	**0.047**	0.147	**0.047**

OCT data (AROC: 0.970)	0.431	0.576	0.879	0.562

Significance (p) values of Area under Receiver Operating Characteristic (AROC) curves were calculated by DeLongs non-parametric method.

SAP data: Standard Automated Perimetry data, based on Pattern Deviation (PD) probability scores

F-SAP data: Fused SAP data, based on weighted transformation of PD probability scores with OCT-derived probability scores

OCT data: Age - and refraction corrected Optical Coherence Tomography A-scan data, optimized by principal component analysis (PCA)

F-OCT data: Fused OCT data, based on weighted transformation of A-scan measurements

with PD probability scores and optimized by PCA

**Bold **indicates statistical significance (i.e. p < 0.05)

The ANN with input based only on the processed OCT A-scan data (AROC: 0.970) had larger AROC compared to the ANN based on PD probability scores (AROC: 0.945). Even though this difference was not statistically significant, the higher performance of the OCT-based ANN prevented any differences from reaching statistical significance when comparing the OCT-based ANN to ANNs trained on the integrated or fused input data. At high specificities fused OCT parameters provided the highest sensitivity values (Figure 3)

**Figure 4:**
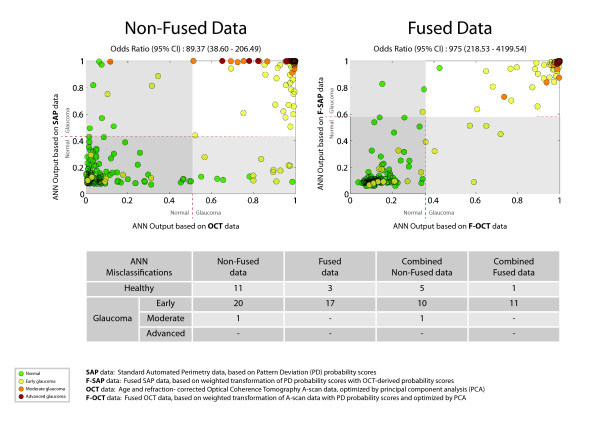
**Artificial Neural Network (ANN) Classification for fused and non-fused data**. The two diagrams show the classification output of the ANNs based on fused and non-fused data, for each healthy individual and glaucoma patient. The odds ratios given for each diagram signify the chance that a test will be classified as normal or abnormal by both SAP-based and OCT-based ANNs. The number of misclassified tests from ANNs based on these different types of data, as well as their combination, is also shown.

**Figure 5:**
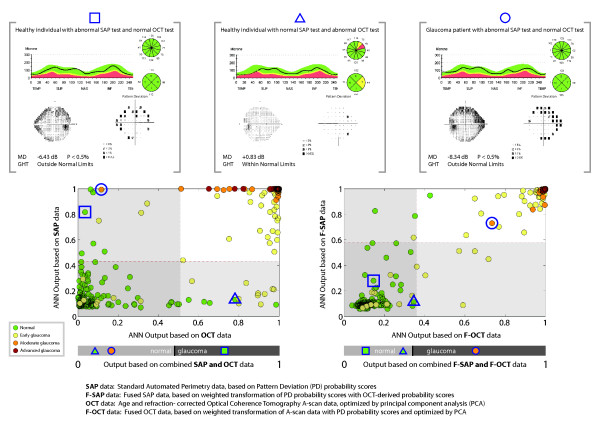
**Classification examples for OCT-based and SAP-based Artificial Neural Networks**. Three examples (two healthy individuals and one glaucoma patient) with disagreement between the SAP-based and OCT-based ANN classification results are highlighted. This disagreement is not evident in the classification results of ANNs based on fused OCT (F-OCT) and SAP (F-SAP) input data, where all three individuals are correctly classified. The ANN classification results for these three persons using combined OCT and SAP as well as combined F-OCT and S-SAP data are also provided under each diagram.

The agreement in classification (reflected by the odds ratios) between ANNs based on SAP and OCT measurements improved when using the fused parameters as input. The improved agreement led to a larger number of individuals correctly classified by both function-and structure-based ANNs (Figure 4). Examples on such classification improvements are given in Figure 5. The missclassified tests belonging to glaucoma patients, did not exhibit discernable visual field or RNFLT defects in neither the SAP nor the OCT tests.

## Discussion

We evaluated the effect of combining SAP and OCT measurements on the ability of ANN classifiers to discriminate between normal and glaucomatous tests. We have previously demonstrated that the use of pre-processed RNFLT measurements based on A-scans improved the diagnostic performance of MLCs compared to the conventional RNFLT parameters presented by the instrument [25]. For SAP, the Pattern Deviation probability plots and maps provide probability values of all test points, highlighting those points with values falling outside the age corrected normal limits and also account for effects of media opacities on light sensitivity across the visual field. The performance benefits of pattern deviation score - based input data have been shown [21].

The combination of structural and functional information contained in the OCT and SAP test data respectively, can be viewed as a type of information integration. The simplest way to integrate the different types of data is to construct a vector that consists of all OCT and SAP measurements. We additionally attempted to construct and evaluate the performance of novel input parameters that fuse both structural and functional measurements. Integrating information about the structure-function relationship of glaucomatous damage through data fusion, presents some advantages over the simple combination of the two different types of data. Instead of relying on MLCs to learn about the structure-function relationship based on limited training data, the fusion process allows for direct incorporation of prior knowledge obtained in other independent large datasets about the topographic relationship between structural and functional measurements into the classification problem. Controlling the incorporation of knowledge into MLCs can also counteract the lack of insight on the way stochastic processes like ANNs represent and use the acquired knowledge in their classification decisions. Our ANNs with input based on the novel parameters showed a high degree of agreement in their classification decisions, reflected on the presented odds ratio values (Figure 4). The higher odds ratios for the ANNs based on fused input data could indicate that these classifiers are more robust since the likelihood of a false positive or false negative test result by both fused OCT and SAP based ANNs was significantly lower.

Bowd et al has previously shown that MLCs trained on combinations of OCT and SAP derived input performed at least as well as MLCs trained on each input type alone, while the use of data with reduced complexity (by means of the backward elimination technique), further improved MLC performance [35]. Our results did not show significant improvement using input that simply combined OCT and SAP measurements compared to when using SAP or OCT measurements separately. However, the combination of fused OCT and SAP parameters showed significant improvement compared to the use of ANNs based on SAP parameters alone, and to the best performing commercially available algorithms in both the SAP and Stratus OCT instruments. This improvement was not specific to our ANN, but could be also seen with another MLC, a relevance vector machine (RVM) classifier, that we constructed and tested for comparison purposes. We did not report the results of our RVM since its performance was very similar to that of our ANN.

The use of principal component analysis for dimensionality reduction of the OCT and fused OCT data instead of a non-linear dimensionality reduction algorithm could have affected the results. Even though non-linear dimensionality reduction techniques might provide better representations of complex data, their extensions to new data are iterative in nature without exact numerical solutions in most cases.

The performance of Machine learning classifiers is dependent on their training process. During training, it is important to present learning examples with a known outcome (i.e. 'true' normal and 'true' glaucoma cases) and with all disease stages in order for the MLC to create representative classification decision boundaries. The inclusion of cases with an uncertain condition (i.e. patients characterized as glaucoma suspects) would adversely affect the false positive and negative rates of classification and our evaluation of specificity and sensitivity rates of the classifier.

The recruitment of healthy persons was based on a random population sample with the majority of individuals having no previous experience in ophthalmic examinations. In our attempt to include healthy individuals that do not represent supernormal subjects, we did not exclude persons with cataract since it is a condition often seen in older population groups and in patients with glaucoma. The rates of missclassifed tests could be partly explained by our choice of reference standard based on ONH morphology, which did not exclude patients with normal SAP and OCT test results. The bias in selecting a structure-or function-related reference standard, affects the accuracy of combinatorial analyses by erroneous estimations of specificity, sensitivity and correlation measures of the examined structural and functional parameters. We did not base the definition of normality and glaucoma on either SAP or OCT test indices. Our choice of reference standard was instead based on clinical examination of ONH morphology. Even though this structure based reference standard relates more to RNFL morphology than function as measured by the visual field, it has not shown a high degree of correlation with OCT measurements [43]. The significant differences in age and refraction between healthy individuals and glaucoma patients are accounted for both in the pattern deviation probability based SAP input and the age-and refraction-corrected OCT input. Even though the 10-fold cross-validation process can account for certain bias pertaining to sample variability, further evaluation on an independent group of subjects is needed to support the general applicability of our findings. Future studies should also evaluate the fusion process with data based on the new generation of spectral domain OCT that provide higher spatial resolution and improved algorithms for detecting and analyzing the RNFL.

The incorporation of knowledge about known rules into black box classifiers could enable the construction of ANN-based systems that are more closely related to grey box models (i.e. models with known general structure but also unknown parameters), allowing for greater insight into the classification process and more effective sensitivity analyses of the test input parameters. Such advantages could facilitate the practical deployment of ANNs as decision support systems in glaucoma diagnostics.

## Conclusions

Our study showed that the combination and fusion of data from OCT and SAP has the potential to increase the accuracy of glaucoma diagnostics compared to parameters from either instrument alone. Moreover, fusion of test measurements could lead to test parameters that better reflect both the structural and functional glaucomatous changes that occur during the course of the disease, providing more relevant information to glaucoma diagnostic systems.

## Competing interests

No conflicts of interest exist between the authors and other parties. AH and BB are both consultants for Carl Zeiss Meditec. All authors (DB, AH, BB) have applied for a patent pertaining to the presented methodology.

## Authors' contributions

DB conceived the study and the study design, collected the study material, conducted programming, performed statistical analyses and drafted the manuscript. AH participated in the study design, its coordination, and aided in drafting the manuscript. BB participated in the study design and its coordination, aided in statistical analyses and in drafting the manuscript. All authors read and approved the final manuscript.

## Pre-publication history

The pre-publication history for this paper can be accessed here:

http://www.biomedcentral.com/1471-2415/11/20/prepub
